# Correction: Transgenic Expression of the Dicotyledonous Pattern Recognition Receptor EFR in Rice Leads to Ligand-Dependent Activation of Defense Responses

**DOI:** 10.1371/journal.ppat.1004872

**Published:** 2015-04-23

**Authors:** Benjamin Schwessinger, Ofir Bahar, Nicholas Thomas, Nicolas Holton, Vladimir Nekrasov, Deling Ruan, Patrick E. Canlas, Arsalan Daudi, Christopher J. Petzold, Vasanth R. Singan, Rita Kuo, Mansi Chovatia, Christopher Daum, Joshua L. Heazlewood, Cyril Zipfel, Pamela C. Ronald

The third author's name is spelled incorrectly. The correct name is: Nicholas Thomas.
