# Supplementary figures and images for: Transgenic Expression of the Dicotyledonous Pattern Recognition Receptor EFR in Rice Leads to Ligand-Dependent Activation of Defense Responses
Source: PLoS Pathog. 2015 Mar 30;11(3):e1004809. doi: 10.1371/journal.ppat.1004809 (PMC4379099; doi:10.1371/journal.ppat.1004809)

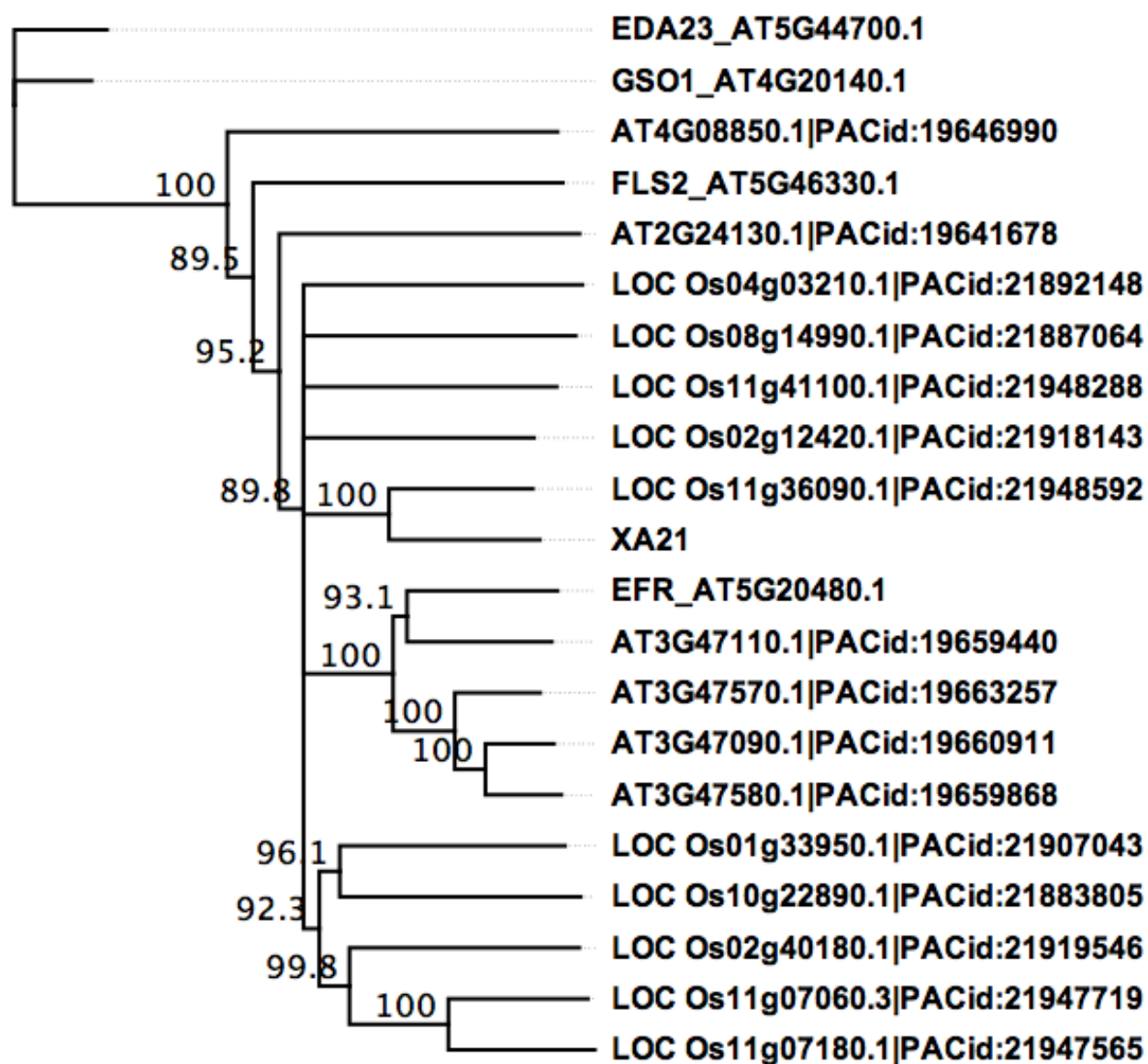

Supplement: S1 Fig — (PDF) [file ppat.1004809.s001.pdf]

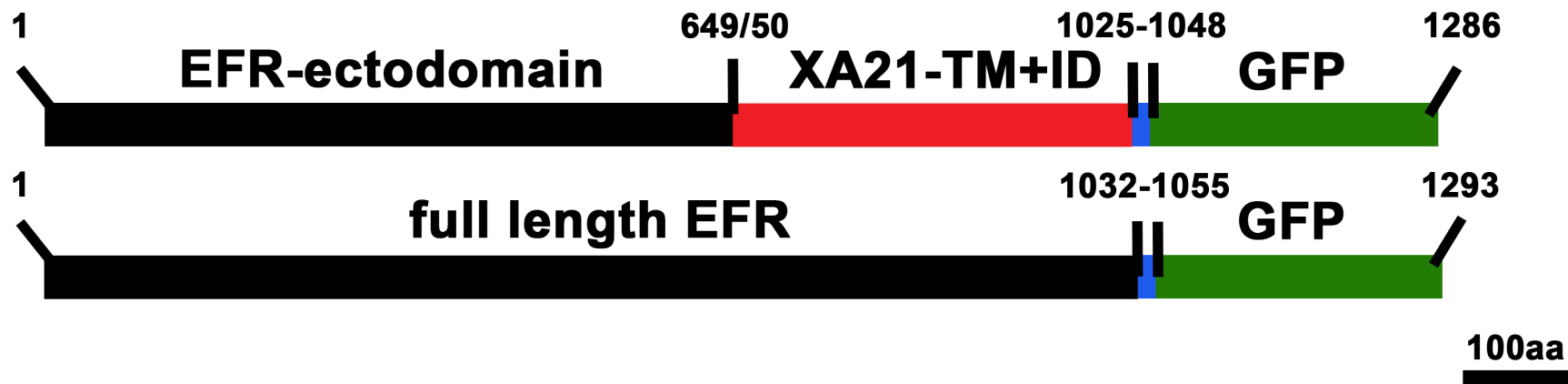

Supplement: S2 Fig — Numbers indicate amino acid residues of fusion points. Drawn to approximate scale. (PDF) [file ppat.1004809.s002.pdf]

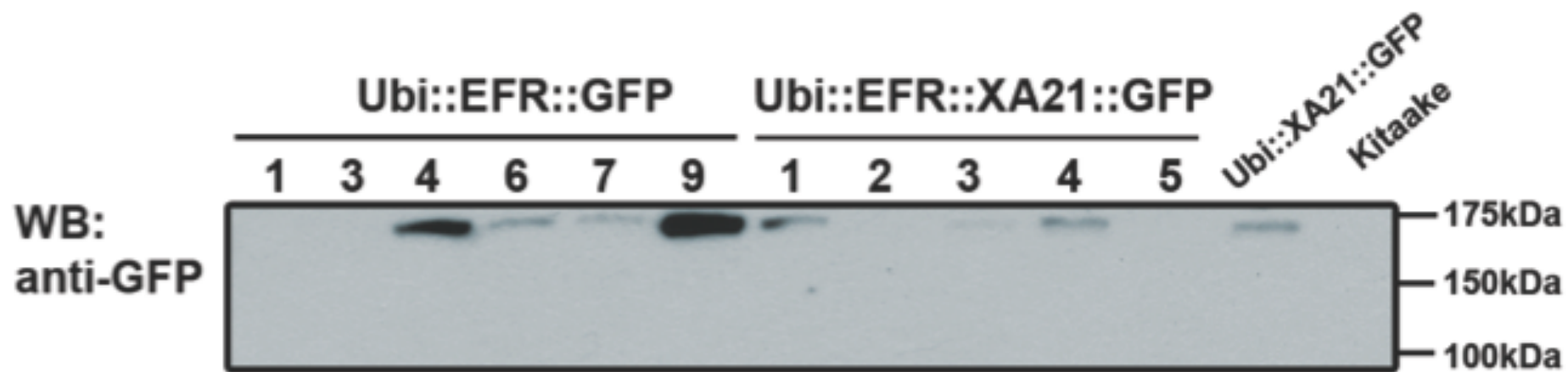

Supplement: S3 Fig — Western blot analysis of pooled total protein fractions of several PCR positive T1 plants for each independent T0 line of Ubi::EFR::GFP and Ubi::EFR::XA21::GFP transgenic rice plants. (PDF) [file ppat.1004809.s003.pdf]

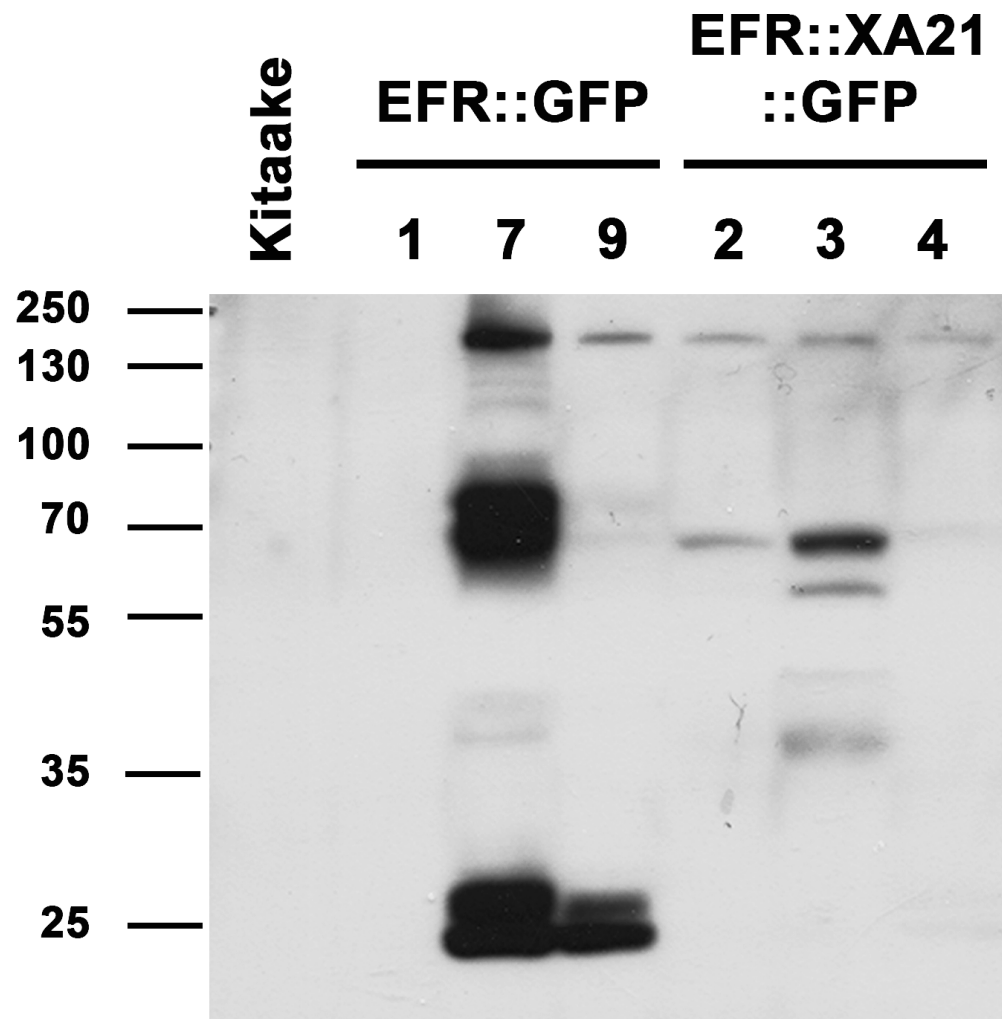

Supplement: S4 Fig — (PDF) [file ppat.1004809.s004.pdf]

**A**

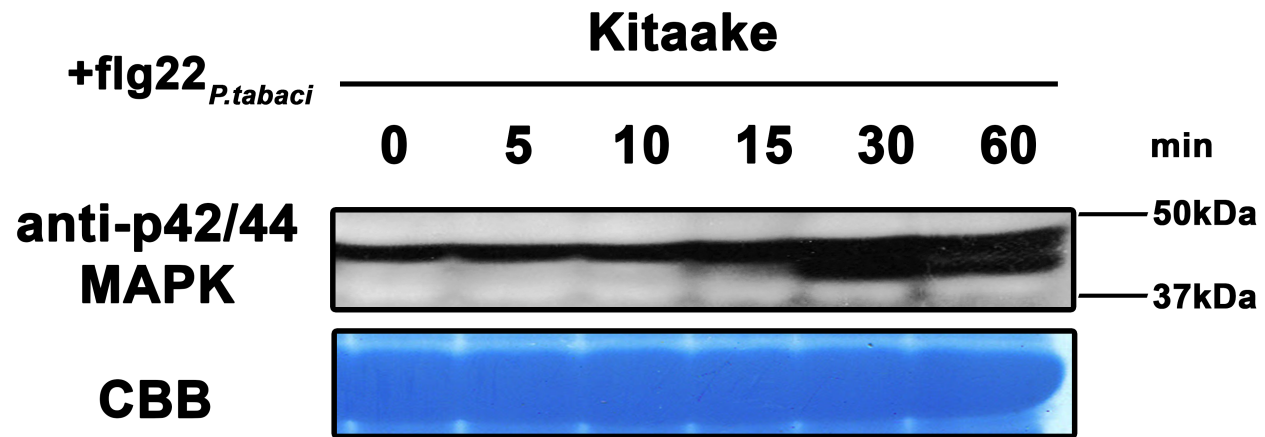

**B**

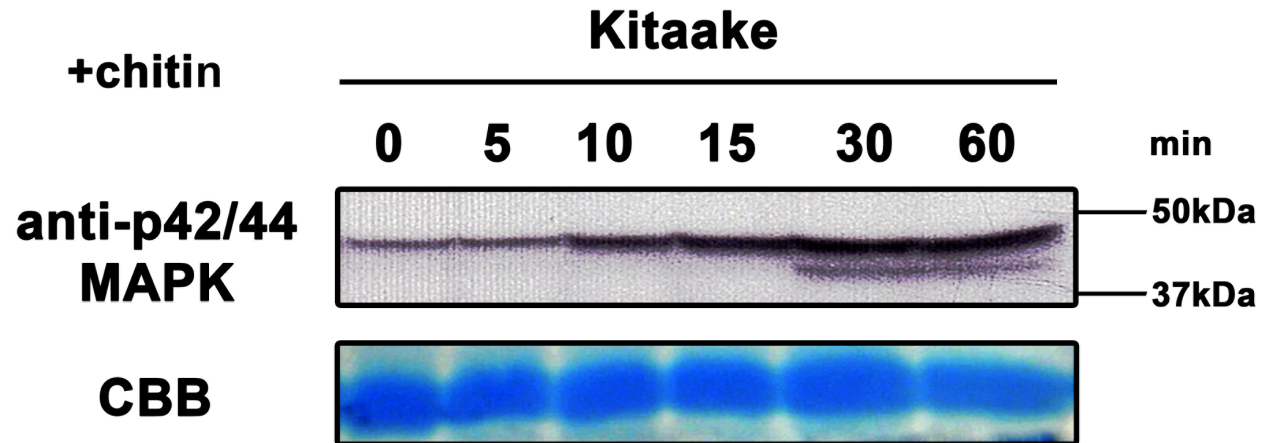

Supplement: S5 Fig — Fully mature leaves of Kitaake were treated with (A) 1 μM flg22Pta or (B) 50 μg/ml chitin for the indicated time. Upper panel anti-p42/44 MAP kinase western blot on total protein extracts, lower panel CBB stain of membrane as loading control. (PDF) [file ppat.1004809.s005.pdf]

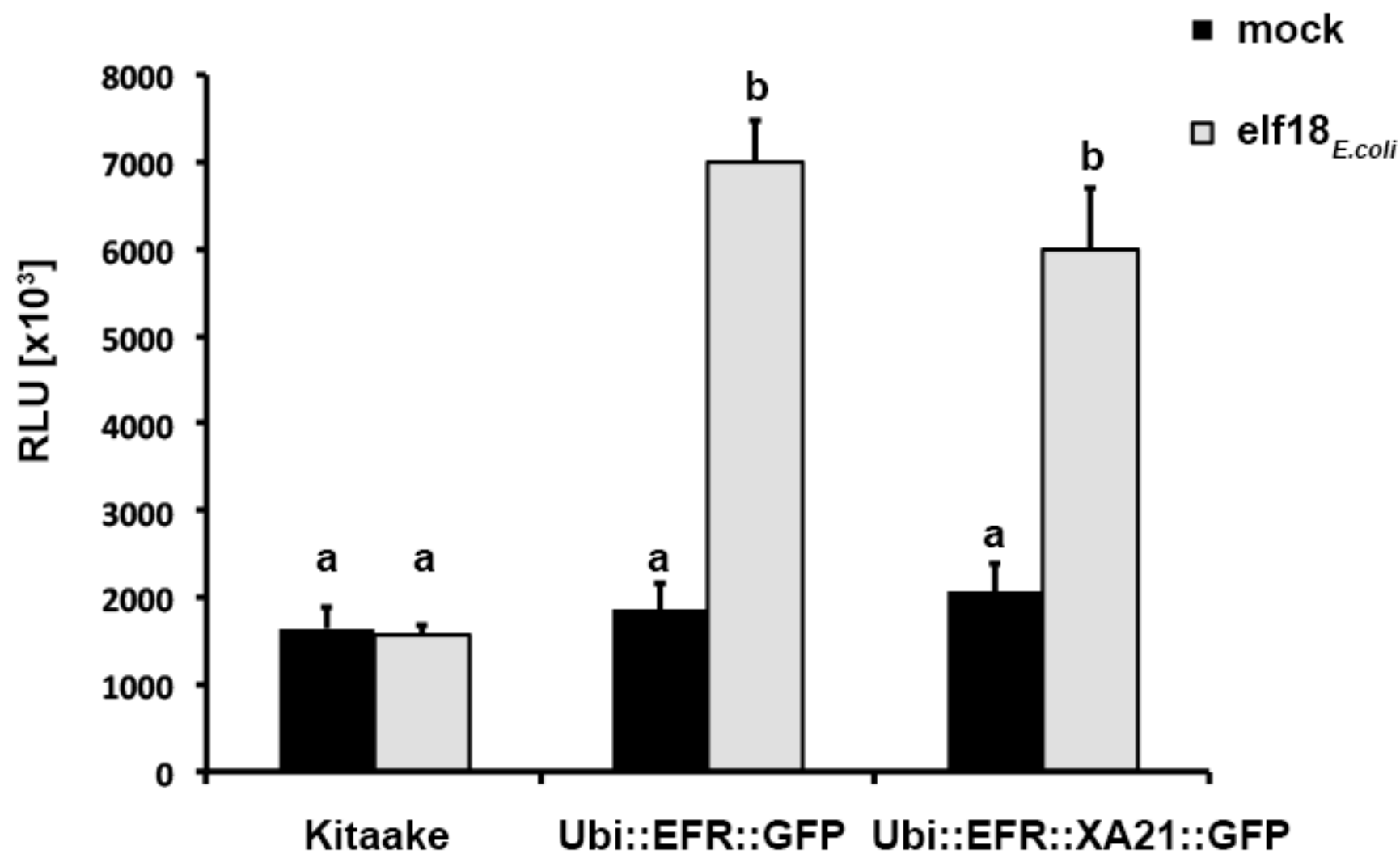

Supplement: S6 Fig — Bars depict average relative light production ± SE of at least six biological replicates. Statistical analysis was performed using the Tukey-Kramer HSD test. Different letters indicate significant differences (p < 0.05). These experiments were repeated at least three times with similar results. (PDF) [file ppat.1004809.s006.pdf]

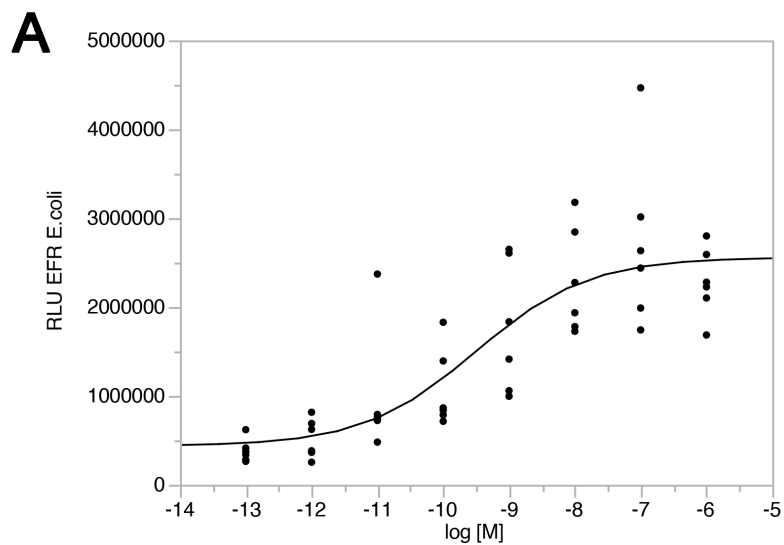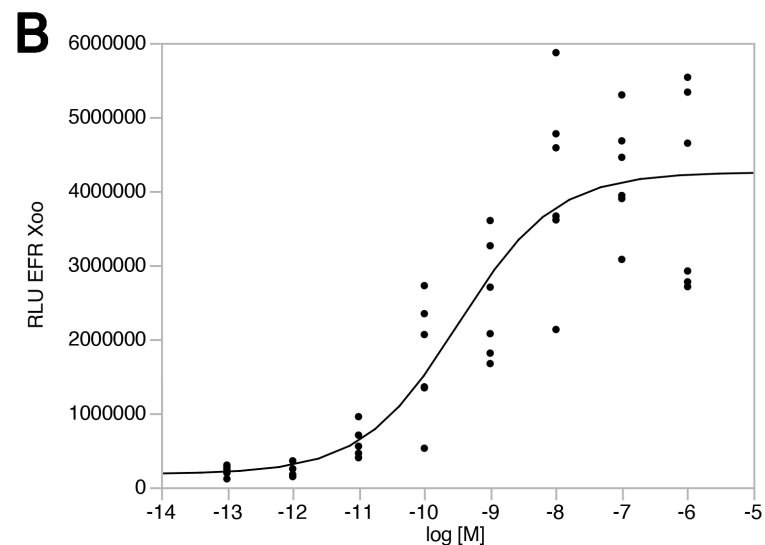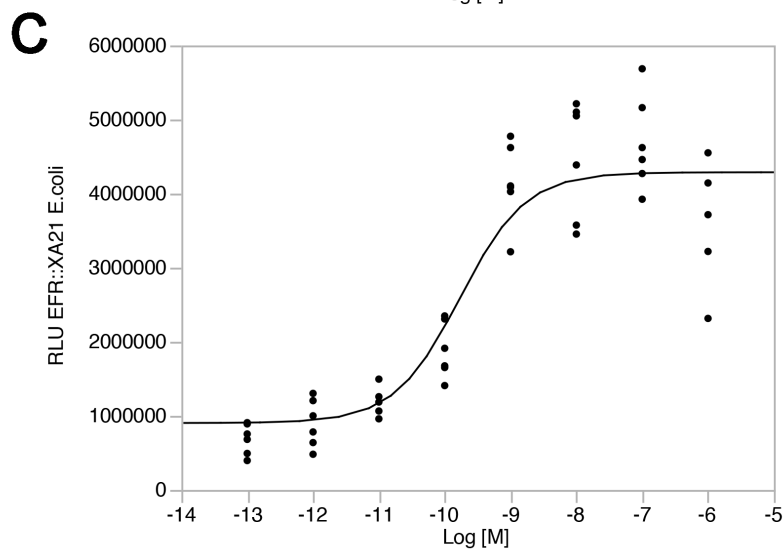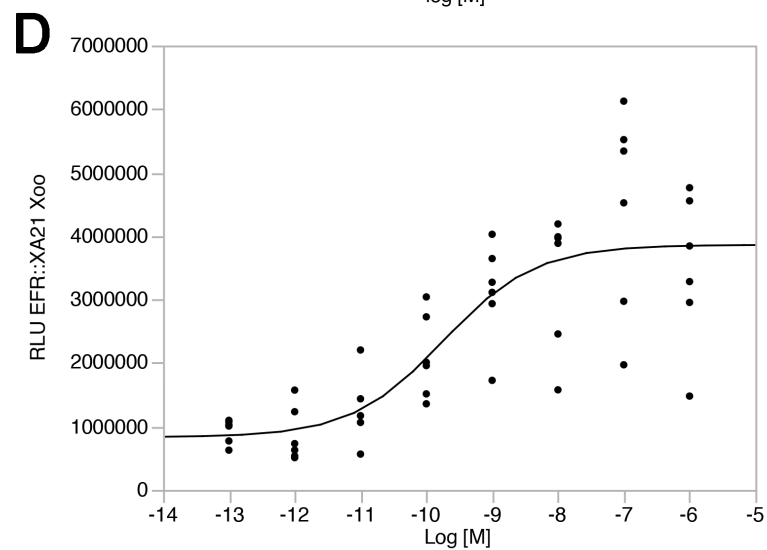

Supplement: S7 Fig — (A) and (B) show dose response curves of EFR::GFP plants in response to the indicated concentrations of elf18E.coli and elf18Xoo, respectively. (C) and (D) show dose response curves of EFR::XA21::GFP plants in response to the indicated concentrations of elf18E.coli and elf18Xoo, respectively. Individual points depict single measurements of total ROS production over 3 hours. The line depicts the best-fitted model to the non-linear logistic 4p formula using the JMP software package. (PDF) [file ppat.1004809.s007.pdf]

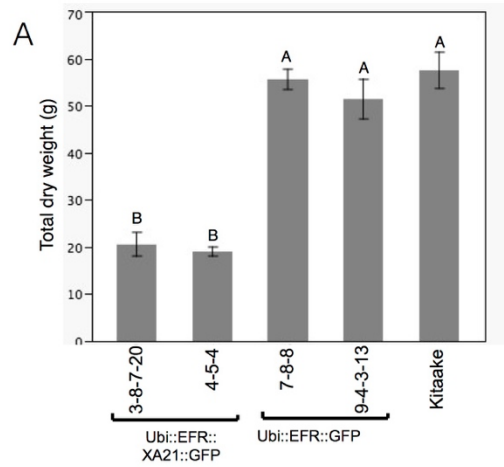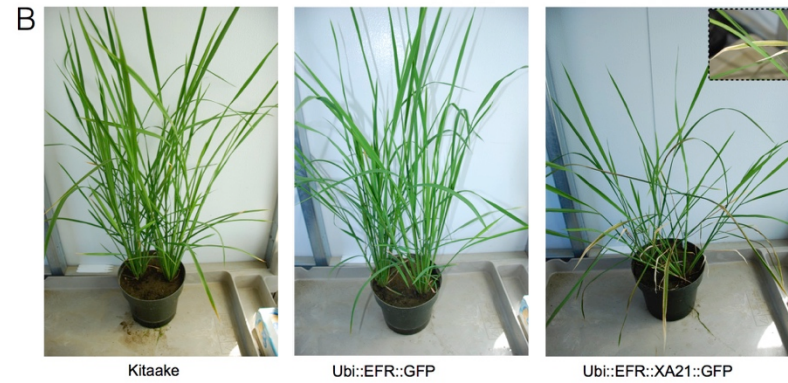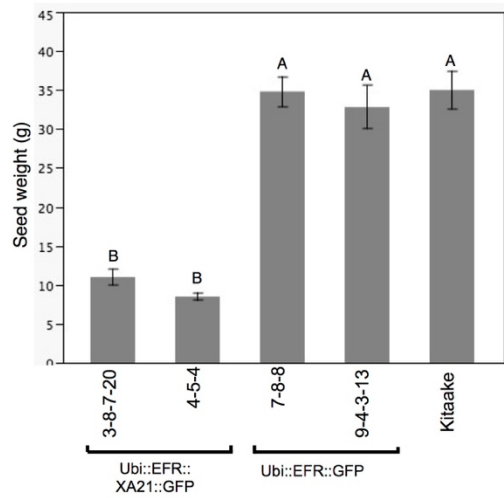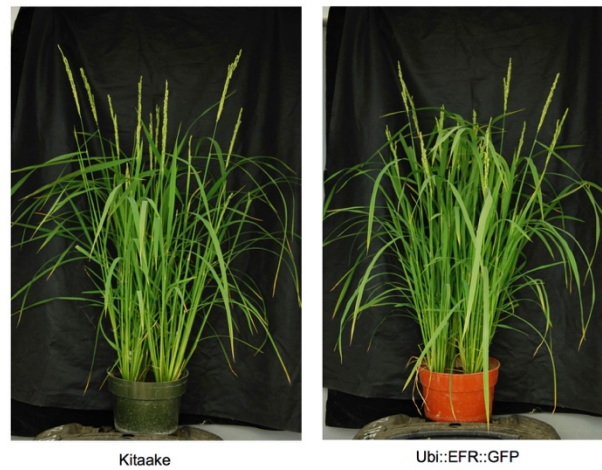

Supplement: S10 Fig — (A) Total dry weight (top) and total yield (bottom) analysis of Kitaake compared with two independent lines of Ubi::EFR::GFP and Ubi::EFR::XA21::GFP transgenic lines. Statistical analysis was done using the Tukey-kramer HSD test. Different letters indicate significant difference at the 0.05 alpha level. (B) Pictured illustration of Kitaake vs. Ubi::EFR::GFP and Ubi::EFR::XA21::GFP lines at the vegetative stage (top), and Kitaake Vs. Ubi::EFR::GFP at the flowering stage. Boxed with dashed line is a zoon-in image of a characteristic necrosis appearing in the Ubi::EFR::XA21::GFP line at 6-week stage. (PDF) [file ppat.1004809.s010.pdf]

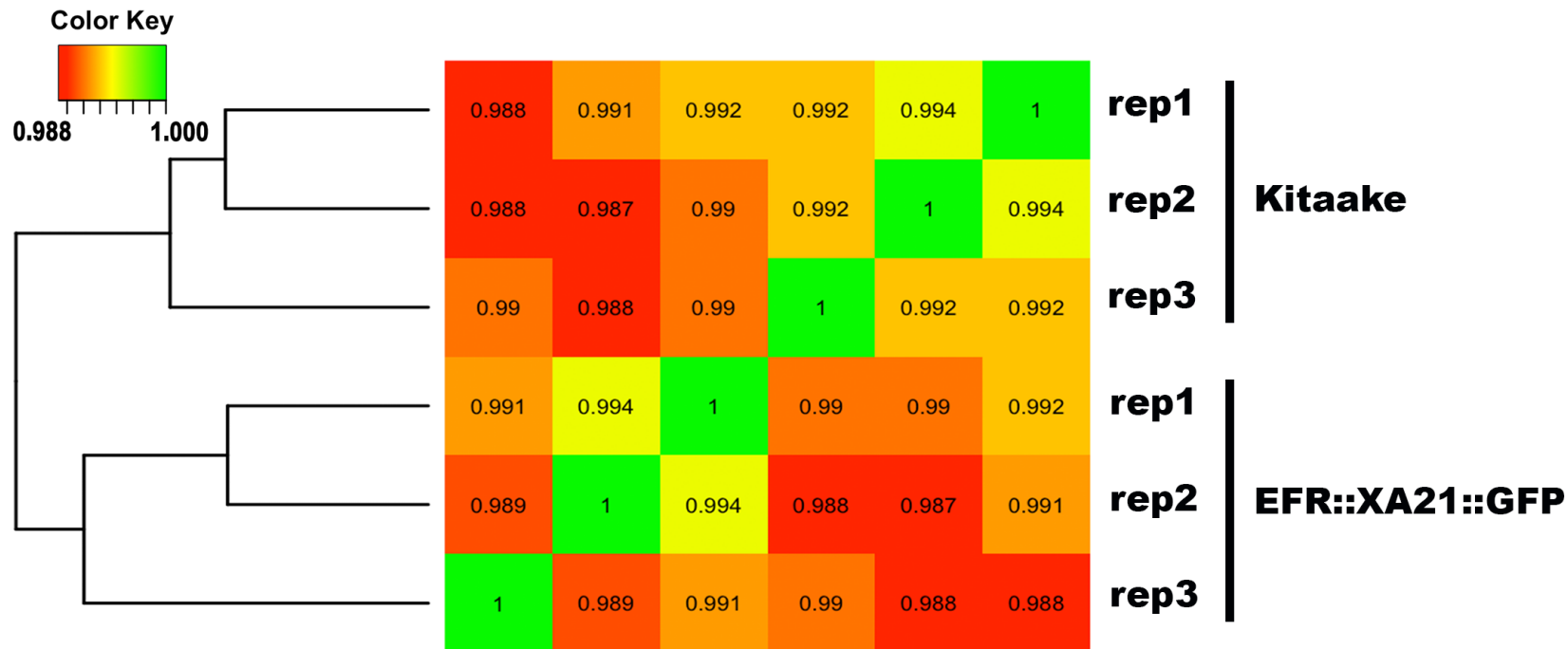

Supplement: S11 Fig — Heatmap and dendogram of Pearson's correlation coefficients between Kitaake and Ubi::EFR::XA21::GFP-3-4. Pearson correlation coefficients were based on logarithmic scaled raw count data. (PDF) [file ppat.1004809.s011.pdf]

A

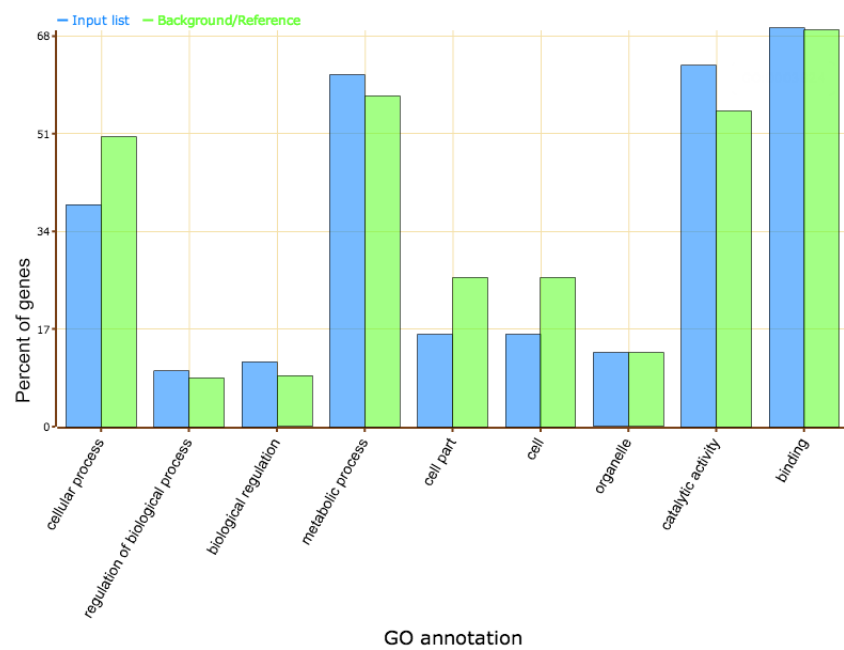

B

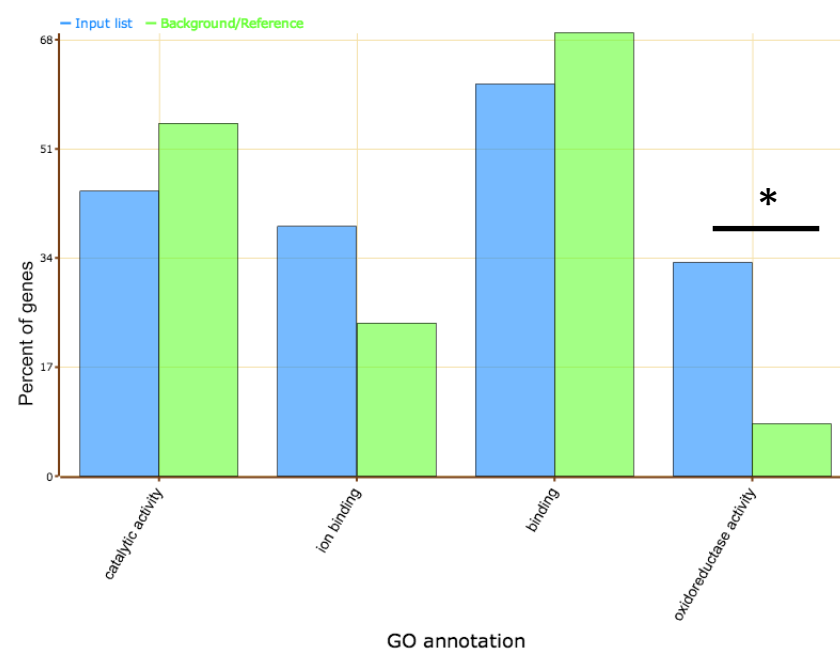

Supplement: S12 Fig — (A) GO terms associated with differentially up-regulated genes between Kitaake and Ubi::EFR::XA21::GFP-3-4 plants. No significant GO term enrichment observed between reference and up-regulated gene set. (B) GO terms associated with differentially down-regulated genes. A significant portion of down-regulated genes is associated with oxidoreductase activity (p = .032, FDR = 0.042). (PDF) [file ppat.1004809.s012.pdf]

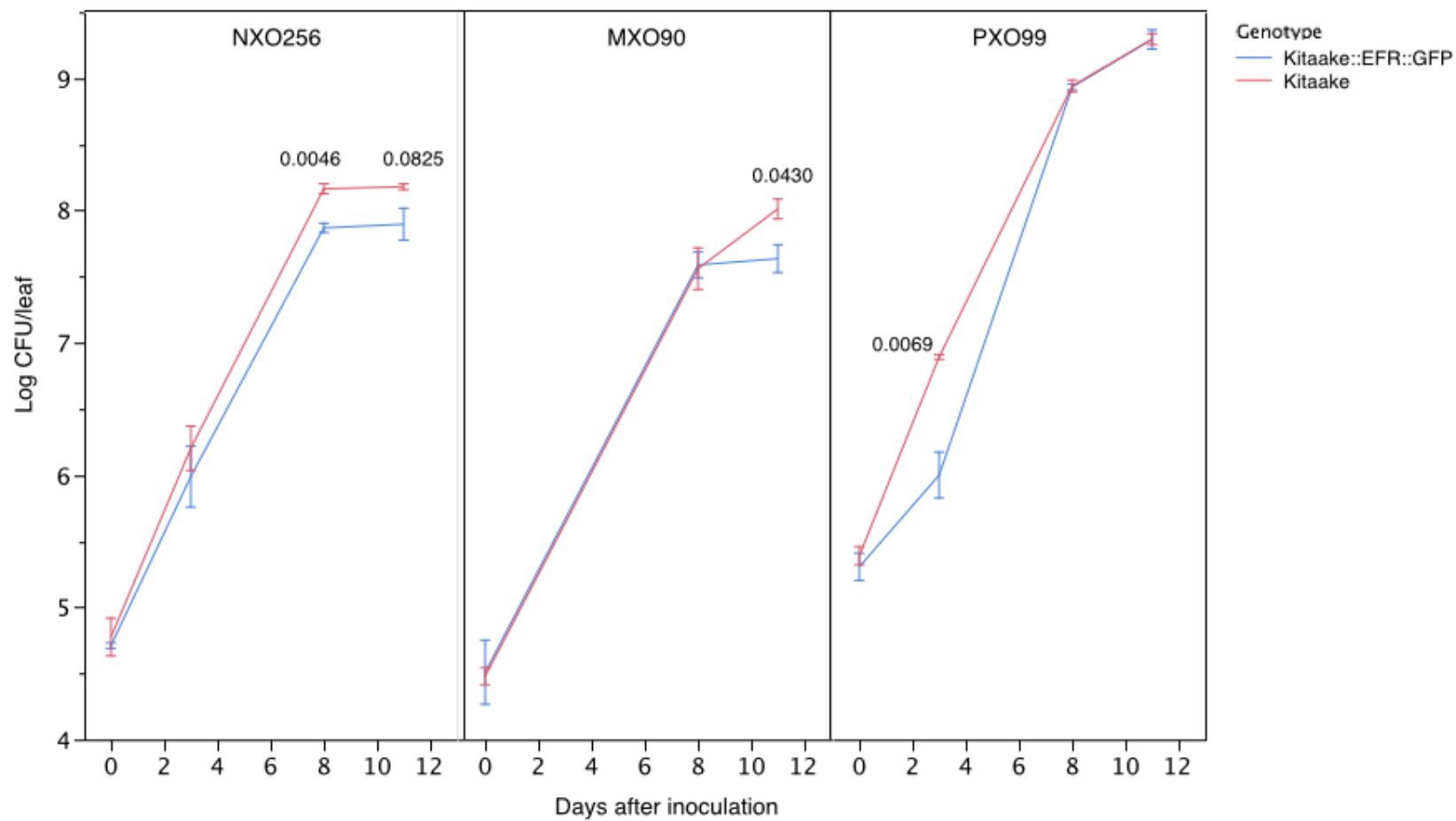

Supplement: S13 Fig — Rice lines were inoculated using the leaf-clipping method as described in materials and methods. Bacterial burden was recorded at 4 time points (0, 3, 8, 12 days post inoculation). Statistical analysis was done using t-test for each time point separately. (PDF) [file ppat.1004809.s013.pdf]

**pBD42**

---

**OssERK2**

**XB3**

**XB15**

**XB24**

**empty**

**XA21**

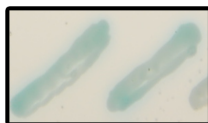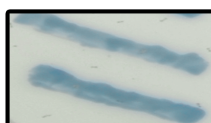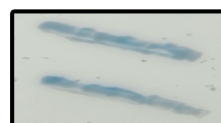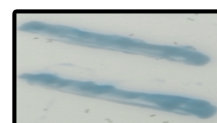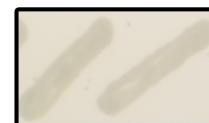

**GUS**

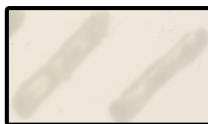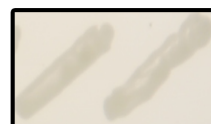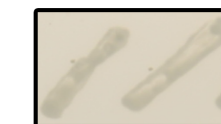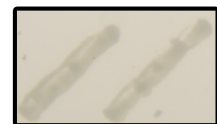

**pLEXA**

Supplement: S14 Fig — Yeast two-hybrid assay between XA21K668 (668-1025aa) and OsSERK2 ID (260-628aa), XB3 full-length (FL) (1-450aa), XB15 FL (1-639aa) and XB24 FL (1-198aa). The blue color indicates nuclear interaction between the two co-expressed proteins. (PDF) [file ppat.1004809.s014.pdf]

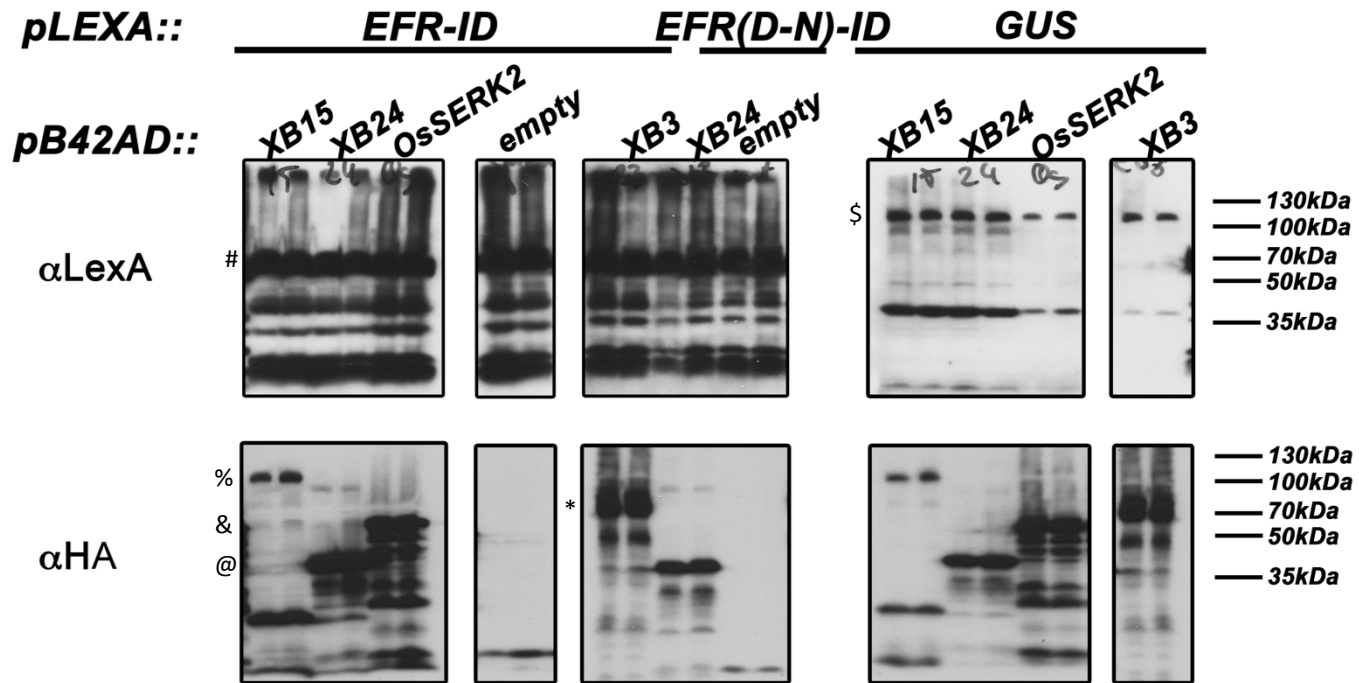

Supplement: S15 Fig — Anti-LexA (upper panel) and anti-HA (lower panel) western blot analysis on total yeast protein extracts from the yeast two-hybrid experiment shown in Fig 5A. # indicates full-length fusion protein LexA-EFR-ID and LexA-EFR(D-N)-ID. $ indicates full-length fusion protein LexA-GUS. % indicates full-length fusion protein AD-XB15. & indicates full-length fusion protein AD-OsSERK2-ID. @ indicates full-length fusion protein AD-XB24. * indicates full-length fusion protein AD-XB3. (PDF) [file ppat.1004809.s015.pdf]

A

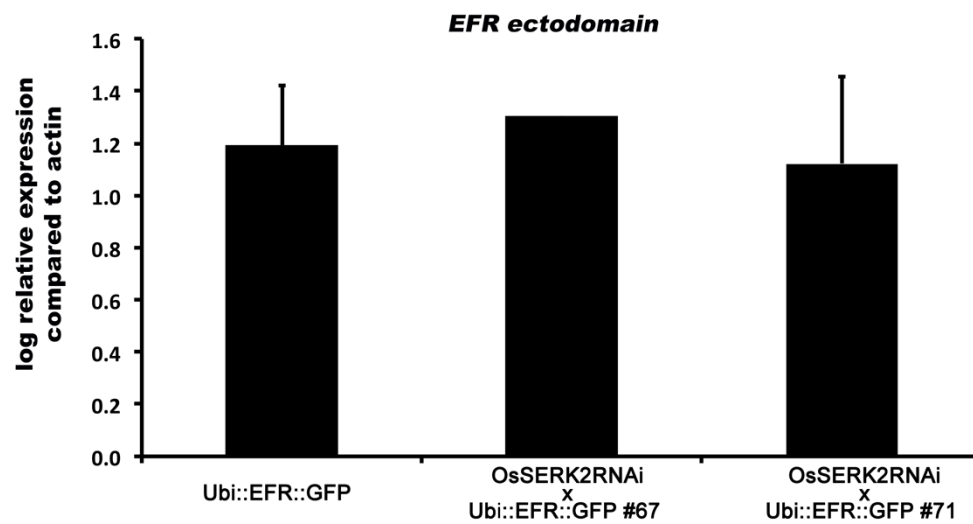

B

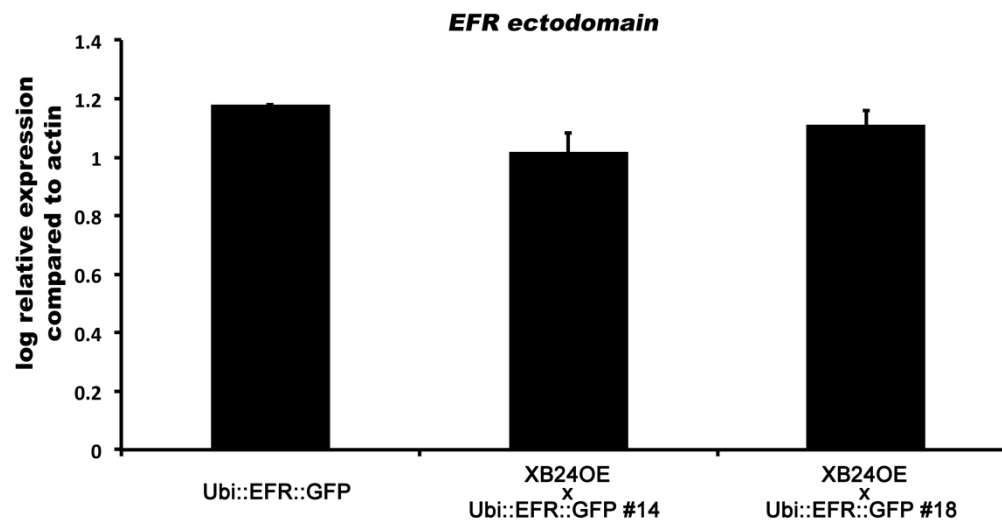

Supplement: S16 Fig — EFR expression in double transgenic lines Ubi::EFR::GFP x OsSERK2RNAi (A) or Ubi::EFR::GFP x XB24OE (B). Expression levels were measured by qRT-PCR and normalized to actin reference gene expression. Bars depict average expression level ± SE of three technical replicates. (PDF) [file ppat.1004809.s016.pdf]

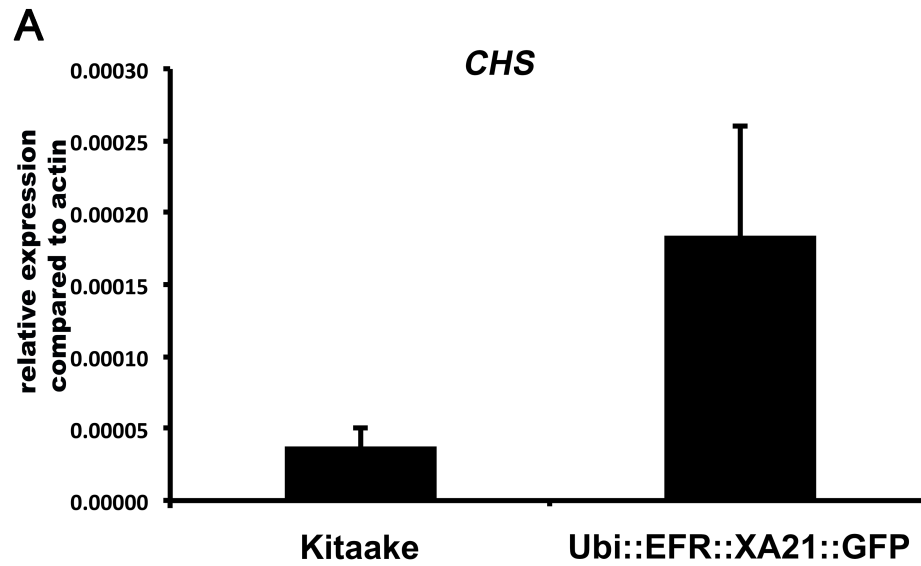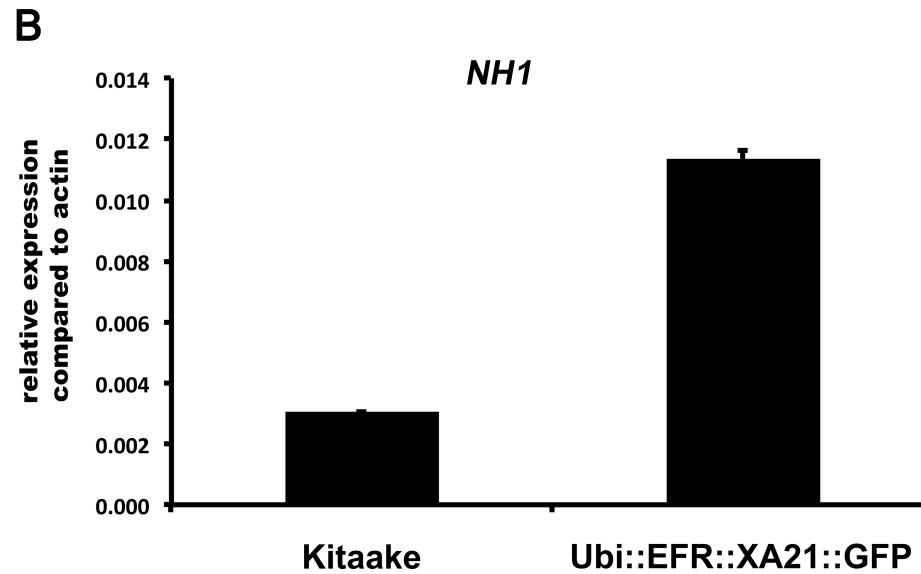

Supplement: S17 Fig — Expression of CHS (A) and NH1 (B) in leaf tissue of 5-week-old Ubi::EFR::XA21::GFP plants. Expression levels were measured by qRT-PCR and normalized to actin reference gene expression. Bars depict average expression level ± SE of three technical replicates. (PDF) [file ppat.1004809.s017.pdf]
