# Supplementary material for: Transgenic Expression of the Dicotyledonous Pattern Recognition Receptor EFR in Rice Leads to Ligand-Dependent Activation of Defense Responses
Source: PLoS Pathog. 2015 Mar 30;11(3):e1004809. doi: 10.1371/journal.ppat.1004809 (PMC4379099; doi:10.1371/journal.ppat.1004809)
Supplement: S8 Fig — The elf18 sequence is marked with black line and the EF-Tu EFa50 region (176–225) is marked with a hatched line. The EF-Tu protein is present in all tested Xoo isolates in two copies. Sequence analysis of the first ~250 amino acid of both copies in 20 Xoo isolates revealed that they are 100% identical therefore only one EF-TuXoo sequence is shown. The first 18 amino acids (elf18) of Xoo contain two base-pair substitutions at positions 2 and 4, as compared with the sequence of E. coli. The 176–225 (EFa50) region has 66% identity between Xoo isolates and A. avenae, while the full-length protein has 83% identity. (PDF) [file ppat.1004809.s008.pdf]

```
Xoo        MAKAKFERTKPHVNVGTIGHVDHGKTTLTAALTKIGAERFGGEFKAYDAI 50
A. avenae  MAKGKFERTKPHVNVGTIGHVDHGKTTLTAAIATVLSAKFGGEAKKYDEI 50
E. coli    MSKEKFERTKPHVNVGTIGHVDHGKTTLTAAITTVLAKTYGGAARAFDQI 50
           *:* ***************************::.: :  :**  : :* *

Xoo        DAAPEEKARGITISTAHVEYESPSRHYAHVDCPGHADYVKNMITGAAQMD 100
A. avenae  DAAPEEKARGITINTAHVEYETANRHYAHVDCPGHADYVKNMITGAAQMD 100
E. coli    DNAPEEKARGITINTSHVEYDTPTRHYAHVDCPGHADYVKNMITGAAQMD 100
           * ***********.*:****::..**************************

Xoo        GAILVCSAADGPMPQTREHILLSRQVGVPHIVVFLNKADMVDDAELLELV 150
A. avenae  GAILVCSAADGPMPQTREHILLARQVGVPYIIVFLNKCDMVDDEELLELV 150
E. coli    GAILVVAATDGPMPQTREHILLGRQVGVPYIIVFLNKCDMVDDEELLELV 150
           ***** :*:*************.******:*:*****.***** ******

Xoo        EMEVRELLSKYDFPGDDTPIIHGSARLALDGDQSEIGVPAILKLVDALDT 200
A. avenae  EMEVRELLDKYDFPGDDTPIVRGSAKLALEGDKGPLGEQAIDKLAEALDT 200
E. coli    EMEVRELLSQYDFPGDDTPIVRGSALKALEGD--AEWEAKILELAGFLDS 198
           ********.:**********::***  **:**        * :*.  **:

Xoo        FIPEPTRDVDRPFLMPVEDVFSISGRGTVVTGRIERGIIKVGDEIEIVGI 250
A. avenae  YIPTPERAVDGAFLMPVEDVFSISGRGTVVTGRVERGIIKVGEEIEIVGI 250
E. coli    YIPEPERAIDKPFLLPIEDVFSISGRGTVVTGRVERGIIKVGEEVEIVGI 248
           :** * * :* .**:*:****************:********:*:*****
```
