# Supplementary material for: Transgenic Expression of the Dicotyledonous Pattern Recognition Receptor EFR in Rice Leads to Ligand-Dependent Activation of Defense Responses
Source: PLoS Pathog. 2015 Mar 30;11(3):e1004809. doi: 10.1371/journal.ppat.1004809 (PMC4379099; doi:10.1371/journal.ppat.1004809)
Supplement: S9 Fig — Western blot analysis with (A) an anti-EF-Tu antibody and (B) mass spectrometry analysis of PXO99 cell-free supernatants reveal that EF-Tu is present in the outer cellular space under in vitro growing conditions in rich media. (PDF) [file ppat.1004809.s009.pdf]

A

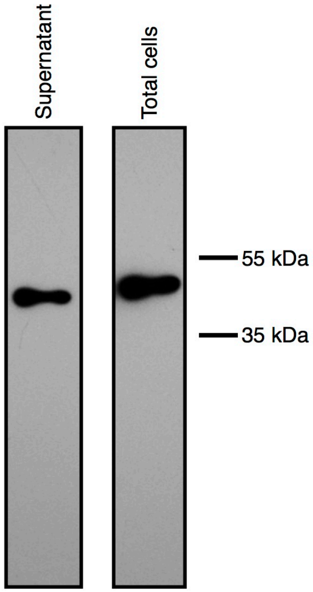

B

fid|24121803|locus|VBIXanOry73153_1278| (100%), 43,080.6 Da
Translation elongation factor Tu [Xanthomonas oryzae pv. oryzae PXO99A]
9 exclusive unique peptides, 10 exclusive unique spectra, 16 total spectra, 125/396 amino acids (32% coverage)

MAKAKFERTK PHVNVGTIGH VDHGK**TTLTA** **ALTK**IGAERF GGEFKAYDAI
DAAPEEKAR**G** **ITISTAHVEY** **ESPSR**HYAHV DCPGHADYVK NMITGAAQMD
GAILVCSAAD GPMPQTREHI LLSRQVGVPH IVVFLNKADM VDDAELLELV
EMEVRELLSK **YDFPGDDTPI** **IHGSARLALD** **GDQSEIGVPA** **ILKLVDALDT**
FIPEPTRDVD RPFLMPVEDV FSISGRGTVV TGRIERGIIK **VGDEIEIVGI**
RATQK**TTVTG** **VEMFR**K**LLDQ** **GQAGDNAGLL** **LR**GTKRDDVE RGQVLCKPGS
IKPHTEFEAE VYVLSKDEGG RHTPFFKGYR PQLYFRTTDI TGAIDLPEGV
EMVMPGDNVK **MTVTLINPVA** **MDEGLR**FAIR EGGRTVGAGV VSKIIK
